# Dengue Virus Infection of the *Aedes aegypti* Salivary Gland and Chemosensory Apparatus Induces Genes that Modulate Infection and Blood-Feeding Behavior

**DOI:** 10.1371/journal.ppat.1002631

**Published:** 2012-03-29

**Authors:** Shuzhen Sim, José L. Ramirez, George Dimopoulos

**Affiliations:** W. Harry Feinstone Department of Molecular Microbiology and Immunology, Bloomberg School of Public Health, Johns Hopkins University, Baltimore, Maryland, United States of America; Washington University School of Medicine, United States of America

## Abstract

The female *Aedes aegypti* salivary gland plays a pivotal role in bloodmeal acquisition and reproduction, and thereby dengue virus (DENV) transmission. It produces numerous immune factors, as well as immune-modulatory, vasodilatory, and anti-coagulant molecules that facilitate blood-feeding. To assess the impact of DENV infection on salivary gland physiology and function, we performed a comparative genome-wide microarray analysis of the naïve and DENV infection-responsive *A. aegypti* salivary gland transcriptomes. DENV infection resulted in the regulation of 147 transcripts that represented a variety of functional classes, including several that are essential for virus transmission, such as immunity, blood-feeding, and host-seeking. RNAi-mediated gene silencing of three DENV infection-responsive genes - a cathepsin B, a putative cystatin, and a hypothetical ankyrin repeat-containing protein - significantly modulated DENV replication in the salivary gland. Furthermore, silencing of two DENV infection-responsive odorant-binding protein genes (OBPs) resulted in an overall compromise in blood acquisition from a single host by increasing the time for initiation of probing and the probing time before a successful bloodmeal. We also show that DENV established an extensive infection in the mosquito's main olfactory organs, the antennae, which resulted in changes of the transcript abundance of key host-seeking genes. DENV infection, however, did not significantly impact probing initiation or probing times in our laboratory infection system. Here we show for the first time that the mosquito salivary gland mounts responses to suppress DENV which, in turn, modulates the expression of chemosensory-related genes that regulate feeding behavior. These reciprocal interactions may have the potential to affect DENV transmission between humans.

## Author Summary

Dengue virus (DENV) is transmitted between humans through the bite of infected *Aedes aegypti* mosquitoes. Since the virus is inoculated in saliva, infection of the mosquito salivary gland is an essential requirement for transmission. In addition, the gland also produces numerous biologically active compounds that facilitate blood-feeding. Despite the salivary gland's crucial role in DENV transmission, very little is known about the host-pathogen interactions, at the molecular level, in this organ. In this study, we characterized the *A. aegypti* salivary gland response to DENV infection at both the gene expression and functional levels. We found that DENV induced the expression of several gene transcripts whose products modulate virus replication in the salivary gland. Unexpectedly, the virus also induced transcripts of two odorant-binding proteins, which we demonstrate to be important for mosquito host-seeking and probing behavior. This is the first study to demonstrate that besides affecting cellular processes that modulate virus replication, DENV also has the potential to alter chemosensory processes in ways that may result in increased virus transmission.

## Introduction

With 2.5 billion people now living in areas at risk for epidemic transmission, dengue has become the most important mosquito-borne viral disease affecting humans [1]. Dengue virus (DENV) is a positive-strand RNA virus of the family *Flaviviridae*, genus *Flavivirus*. It exists as four closely related but antigenically distinct serotypes (DENV-1, -2, -3, and -4), all of which have *Aedes aegypti* mosquitoes as their primary vector, with *A. albopictus* as a secondary vector. The incidence and geographic range of dengue and dengue hemorrhagic fever have increased dramatically in recent decades, and since there is at present no licensed vaccine or drug treatment against DENV, vector control remains the best method for preventing transmission.

Although vertical transmission of the virus has been reported [2], [3], mosquitoes mainly acquire DENV by feeding on the blood of an infected human. DENV first infects and replicates in the mosquito midgut epithelium. It subsequently spreads through the hemolymph to replicate in other organs such as the fat body and trachea, finally infecting the salivary gland at approximately 10–14 days post-bloodmeal [4]. Once in the saliva, DENV can be inoculated into a human host when the mosquito acquires a blood meal, thus spreading the disease.

The mosquito salivary gland plays important roles in DENV transmission. Firstly, infection of the gland itself is an essential part of the transmission cycle. Secondly, the salivary gland produces numerous anti-coagulant, anti-inflammatory and vasodilatory molecules which facilitate probing and bloodmeal acquisition [5]–[10], as well as immune factors that reduce microbial loads in ingested blood and nectar. Lastly, mosquito saliva can impair the immune response of the vertebrate host to arbovirus infection, resulting in increased viremia levels and increasing the risk of virus transmission (reviewed in [11]). Despite its importance in pathogen transmission, the current knowledge on antiviral defense in the salivary gland is limited and is mainly represented by a recent study which identified a cecropin-like peptide with antibacterial and antiviral activities that was induced upon DENV infection of the gland [12].

Mosquitoes are exposed to a variety of microbes in their natural habitats, and possess an innate immune system capable of mounting a potent response against microbial challenge. In addition to RNA interference (RNAi) [13], the Toll and Janus kinase signal transducer and activator of transcription (JAK-STAT) pathways have been found to be key players in *A. aegypti* anti-DENV defense [14], [15]. To date, however, most studies of mosquito antiviral immunity have examined DENV replication in the midgut, but not in other biologically relevant compartments such as the salivary gland. In addition, despite the well-documented involvement of the Toll and JAK-STAT pathways in insect immunity, the specific molecular mechanisms by which these pathways act remain uncharacterized. Viral pathogen-associated molecular patterns (PAMPs) and their associated insect pattern recognition receptors (PRRs) have not yet been discovered, and only a few putative antiviral effector molecules have been identified [12], [15]–[17].

To gain a better understanding of how the *A. aegypti* salivary gland experiences DENV infection at the global transcriptome level, we have used whole-genome microarray-based analyses to compare the naïve and DENV-infected salivary gland. These experiments revealed intriguing patterns of differential transcript abundance that suggested a broad impact of DENV infection on a variety of salivary gland functions, including those implicated in immunity, host-seeking, and blood acquisition. To confirm the functional relevance of DENV-modulated transcript abundance, we used an RNAi-mediated gene silencing approach to show that three DENV infection-induced salivary gland-enriched transcripts can modulate DENV replication in the salivary gland, corroborating the earlier finding [12] that this organ mounts an anti-viral response. In addition, we show for the first time that silencing of two DENV infection-induced odorant-binding protein (OBP) transcripts impaired the host-seeking and blood-feeding ability of mosquitoes, suggesting that the virus is capable of modifying mosquito behavior through the regulation of chemosensory genes. Finally, inspired by these findings, we extended our study to show that DENV is likely to exert a broader impact on mosquito chemosensation by infecting its main olfactory organs, the antennae.

## Results

### The *A. aegypti* salivary gland transcriptome

To determine the *A. aegypti* salivary gland transcriptome in terms of genes whose transcripts are enriched in the uninfected mosquito salivary gland relative to the carcass, we used whole genome microarray analyses to compare transcript abundance in naïve salivary gland and naïve carcass samples. We reasoned that this analysis would yield information about potential gene function, since salivary gland-enriched transcripts would be more likely to perform functions specific to this organ.

**Figure 1:**
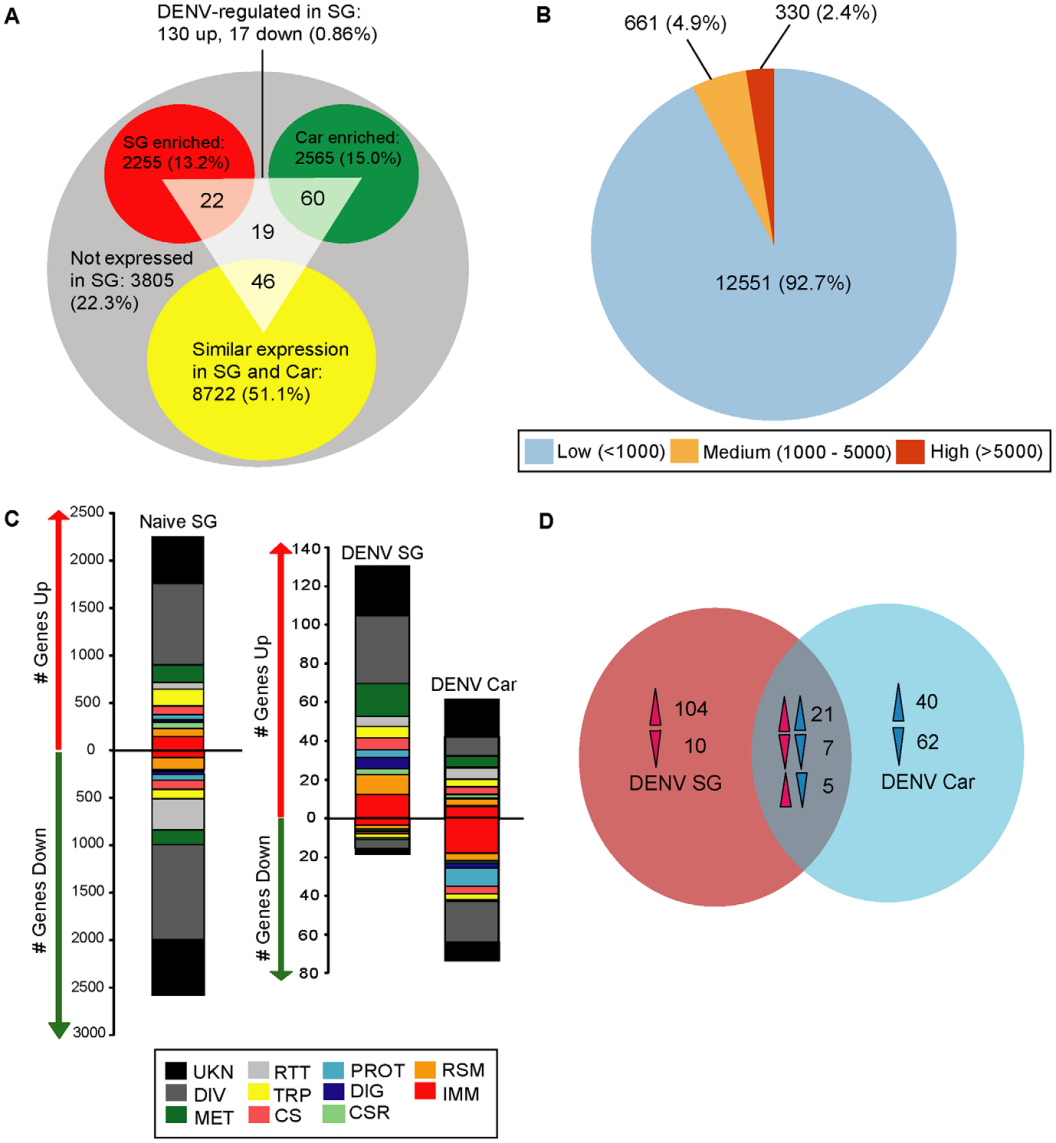
Microarray gene expression analysis of the naïve and DENV-infected salivary gland transcriptomes. (**A**) Numbers of genes that displayed differential (red and green circles) and similar (yellow circle) transcript abundance between the naïve salivary gland and carcass. The superimposed gray triangle shows the numbers of genes displaying significant differences in transcript abundance in the DENV-infected salivary gland, and their overlap with genes expressed in the naïve gland. (**B**) Salivary gland-expressed genes were classified into low, medium and high abundance categories according to their spot intensities on the array. The pie chart shows the number of genes in each category and its corresponding percentage of the total number of expressed genes. (**C**) Functional classification of differentially expressed genes in the naïve salivary gland (relative to the carcass), the DENV-infected salivary gland, and the DENV infected carcass. Functional group abbreviations: CS, cytoskeletal and structural; CSR, chemosensory reception; DIV, diverse functions; DIG, blood and sugar food digestive; IMM, immunity; MET, metabolism; PROT, proteolysis; RSM, redox, stress and mitochondrion; RTT, replication, transcription, and translation; TRP, transport; UKN, unknown functions. (**D**) Venn diagram showing numbers of uniquely and commonly regulated genes in DENV-infected salivary glands and carcasses. Arrows represent the direction of gene regulation.

Of the total number of salivary gland-expressed transcripts, 2255 (13.2%) were significantly enriched in the salivary gland relative to the carcass, 2565 (15.0%) were significantly enriched in the carcass relative to the gland, while 8722 (51.1%) had a similar level of transcript abundance in the two mosquito compartments. The transcripts of 3805 genes (22.3%) were non-detectable or did not meet our signal-to-noise criteria (Figure 1A). Differentially expressed transcripts are presented in Table S1. A previous study by Ribeiro *et al.* (2007) [7] detected transcripts from 835 annotated genes through sequencing of an *A. aegypti* salivary gland expressed sequence tag (EST) library. Our study detected the vast majority (789 out of 835) of these transcripts, supporting the robustness and validity of our microarray-based approach.

Since our microarray-based analyses only provide information on the ratio of differential transcript abundance between the compared samples, we also considered the absolute abundance levels of salivary gland transcripts. Based on the fluorescence intensity of their spots on the microarray, we categorized transcripts into high, medium, and low abundance (Table S2). 330 transcripts (2.4% of the total) were classified as high abundance (fluorescence values >5000), 661 (4.9%) were medium abundance (fluorescence values of 1000–5000), and 12551 transcripts (92.7%) were low abundance (fluorescence values<1000) (Figure 1B). This distribution is comparable to what has been observed for the *Anopheles gambiae* salivary gland transcriptome under the same analysis [6].

Genes that displayed differential transcript abundance between the salivary gland and the carcass represented a range of functional classes (Figure 1C). We next provide a brief description of several functional classes that we consider pertinent to salivary gland function.

#### Sugar and protein digestion

24 transcripts putatively involved in digestive functions were enriched in the salivary gland. Among these were six alpha-amylases (three of which belonged to the high abundance category - AAEL009524, AAEL000392, AAEL006719) and an alpha-glycosidase, which most likely play roles in the digestion of nectar meals. Several protein digestive enzyme transcripts, including 12 trypsins, an amidase, a serine protease and an endopeptidase, were enriched in the salivary gland. These enzymes in saliva could be ingested along with the bloodmeal and aid its digestion. Alternatively, they could be involved in proteolytic events that occur in the vertebrate host during blood-feeding, such as clot prevention or digestion of extra-cellular matrix components [6].

#### Immunity-related functions

148 transcripts with putative immunity-related functions (of which 14 belonged to the high abundance category) were salivary gland-enriched and represented a wide variety of immune gene families. These included two MD2-like proteins, six fibrinogen-related proteins, eight antimicrobial peptides (AMPs), 27 serine proteases, and several Toll pathway-related genes (three Spaetzles and two Tolls). Although nothing is known about the potential roles of these genes in salivary gland immunity, their enrichment in this organ suggests that the gland is capable of mounting potent and diverse immune responses against pathogen challenge to ensure sterility of ingested blood and nectar. AMPs may act against bacteria that the mosquito comes into direct contact with during feeding, for example in sugar sources or on vertebrate skin. One of the salivary gland-enriched AMP transcripts (AAEL000598) encodes a cecropin (AAEL000598) that has previously been found to be induced upon DENV infection in the salivary gland and to possess anti-DENV and antibacterial activities *in vitro* [12]. The large number of salivary gland-enriched serine protease transcripts is striking; these could be implicated in immune pathway activation through the triggering of serine protease cascades, or some of these may play roles in blood-feeding by hydrolyzing host proteins to prevent clotting or inflammation [7].

#### Bloodmeal acquisition

Blood-feeding activates vertebrate host responses that inhibit blood flow, activate antimicrobial defenses, or call the attention of the host to the feeding mosquito. For example, ATP and ADP released from injured cells and activated platelets at the feeding site stimulate platelet aggregation and mast cell degranulation, and adenosine activates inflammatory responses that result in itching and burning sensations, increasing the likelihood of detection of the mosquito [7], [8]. To counteract these responses, mosquito saliva contains numerous enzymes that perform anti-hemostatic and anti-inflammatory roles. Salivary apyrases hydrolyze ATP and ADP to AMP [18]–[20], while adenosine deaminases (ADAs) hydrolyze adenosines to inosines [21]. Three apyrases (two highly abundant - AAEL006347 and AAEL006333) and two ADA transcripts (one highly abundant - AAEL005672) were found to be enriched in the salivary gland.

Thrombin is a key enzyme in the vertebrate blood coagulation cascade. Two putative anti-thrombin transcripts (AAEL007420, AAEL006007) were salivary gland-enriched and also belonged to the highly abundant transcript category. AAEL006007 encodes a Kazal-type serine protease inhibitor that has been partially characterized and found to have anticoagulant and thrombin inhibitory activity [22].

Transcripts of the D7 gene family are widely found in mosquito sialotranscriptomes [7]. D7 protein family members have been suggested to bind and sequester biogenic amines such as serotonin, histamine, norepinephrine and epinephrine [23], which are released at the site of injury and play roles in platelet aggregation, vasoconstriction, and inflammation. Four D7 family member transcripts (one – AAEL006417 – belonged to the highly abundant category) were found to be enriched in the salivary gland. The D7 proteins are related to the odorant-binding proteins (OBPs), and may have been co-opted from this family to scavenge biogenic amines [7], [23].

#### Chemosensory function

62 transcripts with putative chemosensory functions were enriched in the salivary gland, while only 10 were enriched in the carcass, suggesting that the salivary gland may have uncharacterized roles in chemosensory signaling. These transcripts encoded, among other proteins, three putative insect pheromone-binding protein serine/threonine kinases, seven odorant-binding proteins (OBPs), eight gustatory receptors (GRs), and numerous odorant receptors (ORs). The vast majority of these transcripts belonged to the low abundance category, with the exceptions of AAEL006408 (encoding a conserved hypothetical protein with a predicted role in odorant binding) and AAEL002587 (encoding OBP11), which belonged to the high abundance category.

OBPs are small, water soluble, secreted proteins that are highly abundant in the lymph of the sensilla of insect antennae and maxillary palps. They are specialized for ligand binding, and are thought to act as carriers for hydrophobic odorant molecules by facilitating their transport through the aqueous lymph to OR neurons [24], [25]. Enrichment of their transcripts in the salivary gland was somewhat unexpected, but salivary gland OBP transcripts have been reported in *A. aegypti* and *A. gambiae*, and interestingly were found to be transcriptionally down-regulated in this organ after blood-feeding, suggesting that they perform some function related to this behavior [6], [26], [27].

### The DENV infection-responsive *A. aegypti* salivary gland transcriptome

Since DENV replication in the salivary gland is a prerequisite for virus transmission, we next performed a microarray analysis to compare transcript abundance between DENV-infected and naïve salivary glands at 14 days post-bloodmeal (dpbm) to gain a better understanding of how this organ responds to infection. DENV infection stimulated a significant enrichment of 130 and depletion of 17 salivary gland transcripts (Figure 1A, 1C, Table S3).

DENV altered the abundance of 38 transcripts with functions related to metabolic processes, transport and stress response. The majority of these transcripts (33 of 38; 87%) were enriched in the infected salivary gland, perhaps indicating a shift in cellular metabolic state to support virus replication. Six transcripts with predicted cytoskeletal functions were enriched upon infection; this may reflect maintenance in structural integrity of the infected salivary gland, since cytopathology has been reported in this organ following arbovirus infection [28], [29]. Also up-regulated were two tetraspanin transcripts, which encode transmembrane proteins that have roles in cell-cell interactions, adhesion, motility, and proliferation. Tetraspanins have been found to be induced upon DENV infection of *Aedes albopictus* C6/36 cells [30], and are believed to facilitate cell-to-cell spread of virus. Twelve transcripts with immune-related functions were induced by DENV infection, and included two MD2-like gene family members, which code for secreted proteins containing Niemann-Pick lipid recognition domains. Mammalian MD2 is a co-receptor that is required for Toll-like receptor 4 (TLR4) binding to lipopolysaccharide (LPS) [31], [32], and silencing of the *A. gambiae* MD2-like family member AgMDL1 significantly increases midgut *Plasmodium falciparum* infection levels [33]. These data suggest a potential role for *A. aegypti* MD2-like family members in immune defense against DENV.

Transcripts encoding a transferrin and a fibrinogen-related protein were also up-regulated. Transferrins bind iron with high affinity and play roles in iron metabolism, immunity, and development. They are up-regulated upon parasite or bacterial infection, and may sequester iron from pathogens; alternatively, proteolytic fragments from these proteins have also been suggested to also act as anti-microbial peptides or inducers of the immune response [34]. Fibrinogen-related proteins bind bacteria and parasites in mosquitoes and may function as pattern recognition receptors [35].

Transcripts of three leucine-rich repeat (LRR)-containing proteins and one ankyrin repeat-containing protein were induced by DENV infection. The broader LRR-containing protein family includes the mosquito Tolls, and family members are commonly involved in protein-protein interactions and signal transduction pathways [36]. Ankyrin repeats mediate protein-protein interactions and are present in several immune-related proteins, such as the IkB inhibitory domain of the NFkB-like transcription factor Rel2 of the mosquito IMD immune pathway.

Three cathepsin B transcripts and a putative cystatin transcript were induced upon DENV infection. Cathepsin Bs are lysosomal cysteine proteases known to be involved in the apoptosis of immune cells [37]. They can also play roles in TLR signaling, and are required to cleave the endolysosomal TLRs 7 and 9 before these molecules can signal [38]–[40]. Cystatins are cysteine protease inhibitors that may play roles in regulating apoptosis, since many enzymes (such as the caspases and cathepsins) involved in apoptotic pathways are cysteine proteases [41], [42]. A cystatin has also been reported to induce autophagy in mammalian cells [43], and DENV is known to induce autophagy as a means of regulating lipid metabolism in the host cell [44], [45].

The transcripts of three peptides with sequence similarity to secreted salivary peptides from Culicine mosquito species were also up-regulated by DENV infection. The functions of these peptides remain unknown, but some may be involved in the production of allergic reactions to mosquito bites [7].

Finally, DENV also induced two OBP transcripts (OBP10 and OBP22), which had also been found to be enriched in the naïve salivary gland.

To determine whether our observed salivary gland transcriptomic infection responses were specific for this organ, or if they also occurred in other tissues, we went on to characterize the DENV infection-responsive carcass transcriptome at 14 dpbm. DENV infection significantly up-regulated 61 transcripts and down-regulated 74 in the carcass compartment (Figure 1C, Table S4). Only 28 genes were similarly regulated between the salivary gland and the carcass upon infection (Figure 1D), indicating that the transcriptomic responses in these two compartments are quite distinct.

### Functional analysis of selected DENV infection–responsive salivary gland genes

Our transcriptomic analyses suggested that at least some of the DENV infection-responsive transcripts may play roles in limiting infection, or reflect a virus-mediated modulation of salivary gland functions that could have implications for mosquito behavior. We were particularly interested in modulators of DENV replication in the salivary gland. Based on our transcriptomic analyses and literature searches, we selected seven candidate genes for functional analysis via RNAi-mediated gene silencing (Table 1). We have elaborated on the potential modes of action of these genes in the previous section.

**Table 1:** Candidate genes selected for functional testing via RNAi-mediated gene knockdown.

Gene ID	Description	Functional Group	Log_2_-fold			Comment	Tested for role in	
			DENV SG	DENV Car	Naïve SG		DENV replication	Feeding behavior
AAEL015136	Niemann-Pick Type C-2, putative (MD6)	IMM	1.60	−1.19	−1.89	MD2-like, lipid-binding domain	X	
AAEL009760	Niemann-Pick Type C-2, putative (MD21)	IMM	2.12	0.92	−3.59	MD2-like, lipid-binding domain	X	
AAEL014906	LAP4 protein, putative	IMM	0.87		0.81	Leucine-rich repeats	X	
AAEL007585	cathepsin B	IMM	1.49			Lysosomal cysteine protease; pro-apoptotic, role in TLR signaling	X	
AAEL003728	conserved hypothetical protein; ankyrin repeats	DIV/UNK	0.87		0.92	Ankyrin repeats present in several immune-related proteins	X	
AAEL017380	hypothetical protein; putative glycine-rich salivary secreted peptide	DIV/UNK	1.07	1.68	1.94	Putative allergen/anti-coagulant	X	
AAEL013287	conserved hypothetical protein; putative cystatin	DIV/UNK	0.81			Cysteine protease inhibitor, pro-apoptotic, induces autophagy in mammalian cells	X	
AAEL005772	Odorant-binding protein 99c, putative (OBP22)	CSR	1.38		0.86	Putative roles in olfaction		X
AAEL007603	Odorant-binding protein 56a, putative (OBP10)	CSR	0.92		1.22	Putative roles in olfaction		X

Candidate genes selected for functional testing via RNAi-mediated gene knockdown and their associated log_2_-fold values in the DENV-infected salivary gland (DENV SG), DENV-infected carcass (DENV Car), and naïve salivary gland (naïve SG). Functional group abbreviations: IMM, immunity; DIV, diverse functions; UNK, unknown functions; CSR, chemosensory reception.

Mosquitoes were orally infected with DENV through a bloodmeal, and candidate genes were silenced in the salivary gland at 7 dpbm by the injection of 2 ug of dsRNA per mosquito [19]. At this time point, the midgut and carcass are fully infected, and the virus is initiating infection of the salivary gland [4]. Gene silencing efficiency ranged from 26–90% (Figure S1). Salivary glands were subsequently dissected at 7 days post-silencing (14 dpbm), and virus titers were determined by plaque assay.

**Figure 2:**
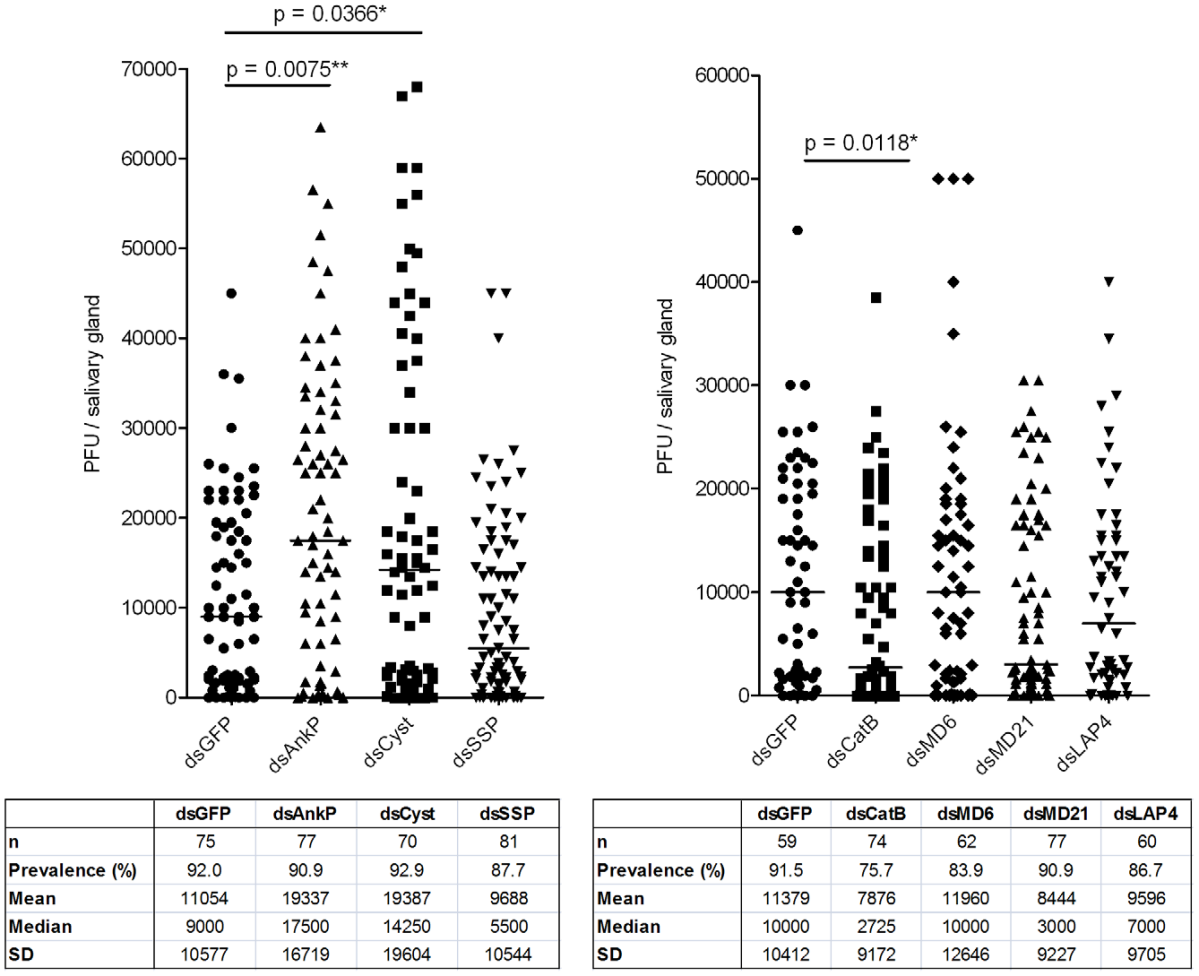
Effect of candidate gene knockdown on salivary gland DENV titers. Candidate genes were silenced in DENV-infected mosquitoes, and salivary gland virus titers at 14 dpbm were determined by plaque assay. A pool of three biological replicates is shown, and p values were determined using the Mann-Whitney U test (*, p<0.05). Gene name abbreviations: AnkP, ankyrin repeat-containing protein; Cyst, cystatin; SSP, salivary secreted peptide; CatB, cathepsin B.

Silencing of the putative cystatin (AAEL013287) and the conserved hypothetical protein with ankyrin repeats (AAEL003728) genes significantly increased salivary gland DENV titers, while silencing of the cathepsin B (AAEL007585) gene resulted in significantly reduced DENV titers (Figure 2).

Since injection of dsRNA into the mosquito thorax results in non-compartment-specific silencing, it is possible that the altered virus titers observed in the salivary gland are a consequence of gene silencing in other parts of the mosquito carcass. However, we consider this less likely for several reasons: firstly, DENV infection induced these genes only in the salivary gland and not in the carcass (Table 1), suggesting that they play infection-related functions in the gland; secondly, dsRNA injections were carried out at 7 dpbm, when carcass DENV titers have already peaked, while salivary gland infection is just beginning [4]; and lastly, we found no significant differences in virus titers between the carcasses of DENV-infected gene-silenced and control GFP dsRNA-treated mosquitoes (Figure S2).

### Effect of OBP gene silencing on mosquito blood-feeding behavior

Transcripts of OBPs 10 and 22 (AAEL007603 and AAEL005772) displayed an elevated abundance in the salivary gland upon DENV infection, and were also enriched in the naïve gland. This finding was unexpected and intriguing to us, and we hypothesized that these genes could participate in chemosensory signaling during host-seeking or probing. To test this hypothesis, these genes were individually silenced by the injection of 2 ug dsRNA per mosquito, 4 days prior to a behavioral feeding assay. Mosquitoes were offered an anesthetized Swiss Webster mouse, and the following parameters were measured: a) Probing propensity (percentage of mosquitoes that probed within a fixed time period); b) Probing initiation time (time from the introduction of the mouse to the time at which the mosquito initiated probing – a rough measure of host-seeking ability); and c) Probing time (time from the initial insertion of the proboscis in the skin to the initial engorgement of blood [6], [10]).

**Figure 3:**
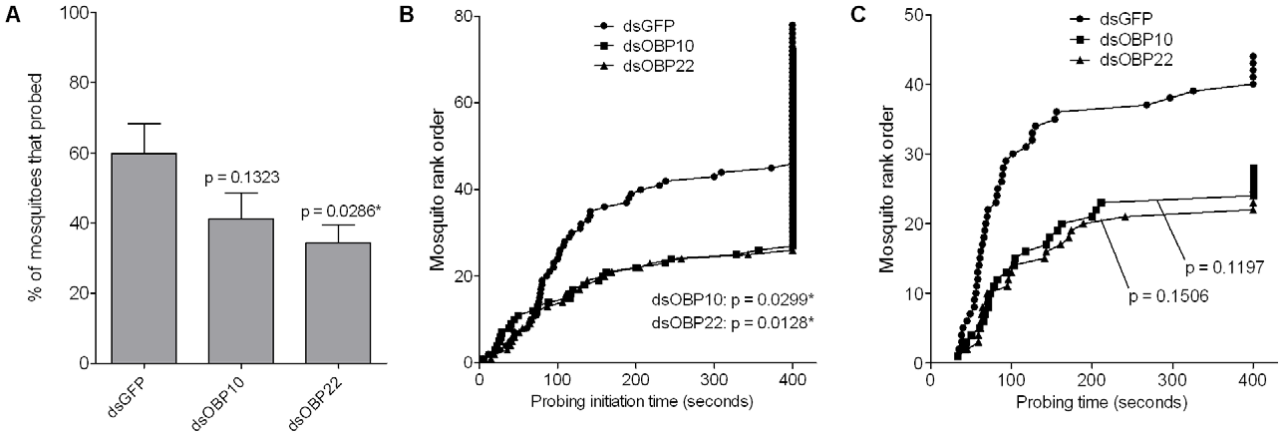
Effect of OBP gene knockdown on mosquito blood-feeding behavior. A behavioral feeding assay was performed 4 days post-silencing of OBPs 10 and 22. Mosquitoes were offered an anesthetized mouse and observed individually for 400 seconds. The following parameters were measured and are represented here: (**A**) Probing propensity: the percentage of mosquitoes that probed within the observation period; (**B**) Probing initiation time: the time from the introduction of the mouse until the mosquito starts to probe; (**C**) Probing time: the time from the initial insertion of the mouthparts in the skin to the initial engorgement of blood. The data are a pool of six biological replicates, and p values were determined with the Mann-Whitney U test (*, p<0.05).

Silencing of the OBP10 or OBP22 genes resulted in a reduced probing propensity, which was statistically significant for OBP22-silenced mosquitoes (Figure 3A). Knockdown of either OBP was found to significantly increase the probing initiation time compared to GFP dsRNA-treated mosquitoes (Figure 3B). Probing time was also increased in OBP gene-silenced mosquitoes, although this increase was not statistically significant (Figure 3C). Since only mosquitoes that probed were considered for the probing time analysis, the lower number of mosquitoes that probed in OBP-silenced groups could have contributed to the lack of statistical significance for this parameter. Taken together, these data indicate that gene silencing of these OBPs impairs the efficiency of mosquito blood-feeding.

### Quantification of OBP transcript abundance and DENV infection in the mosquito chemosensory apparatus

**Figure 4:**
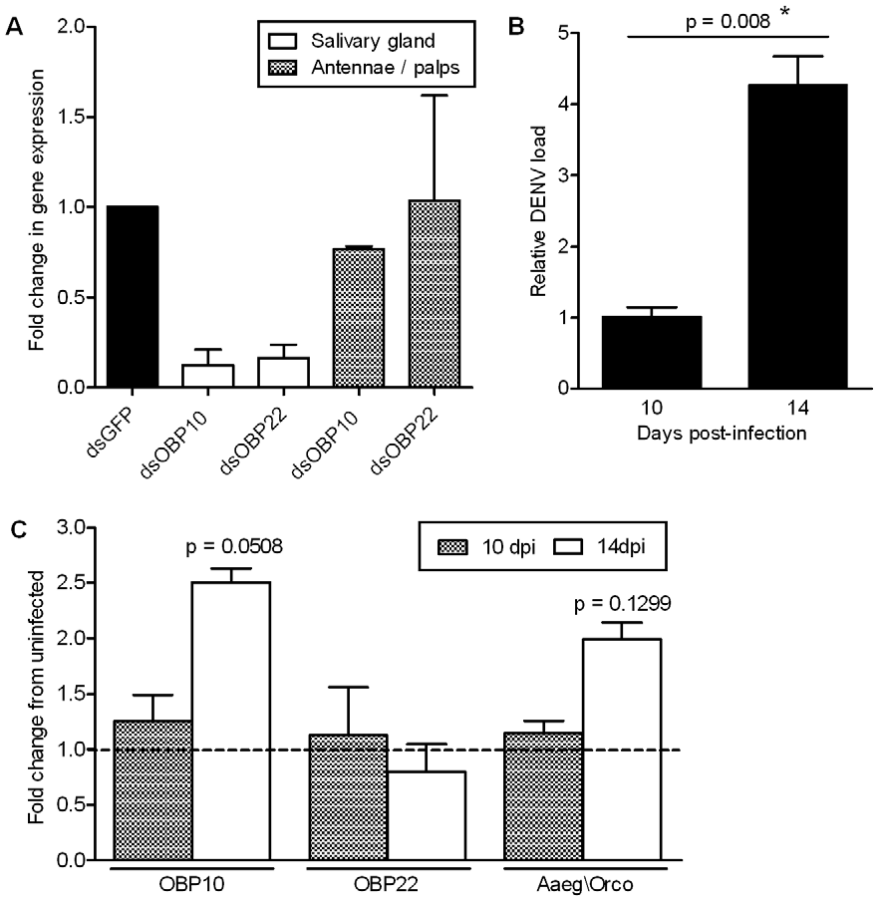
OBP silencing efficiency and gene expression in the chemosensory organs; detection of DENV by RT-PCR in the chemosensory organs. (**A**) Silencing efficiencies for OBPs 10 and 22 in the salivary gland and chemosensory organs (antennae and palps) 4 days post-injection of 2 ug dsRNA, relative to dsGFP-injected controls. (**B**) Detection of DENV by RT-PCR in the antennae and palps of DENV-infected mosquitoes at 10 and 14 dpbm. *, p<0.05 in Student's t-test. (**C**) OBP and Aaeg\Orco gene expression in the antennae and palps of DENV-infected mosquitoes at 10 and 14 dpbm, relative to mock-infected mosquitoes. p values were determined with the Student's t-test.

The observed effect on feeding behavior could also be due to gene silencing in the chemosensory organs (antennae and maxillary palps) instead of or in addition to the salivary gland. To further investigate the molecular basis of this interesting phenotype, we determined OBP gene silencing efficiency in these two body compartments by quantitative RT-PCR. High silencing efficiencies for both OBP genes were consistently obtained in the salivary gland (averages of 87.4% and 86.8% respectively), while efficiencies were lower and more variable in the antennae and palps (average of 22.9% for OBP10; 0%, 73.4%, and 32.5% for three trials of OBP22 (Figure 4A)). While these data suggest that the impaired feeding behavior was at least in part due to OBP gene silencing in the salivary gland, we also considered the possibility that the altered host-seeking and feeding behavior was due to DENV infection of the antennae and its effect on OBP transcript abundance there.

**Figure 5:**
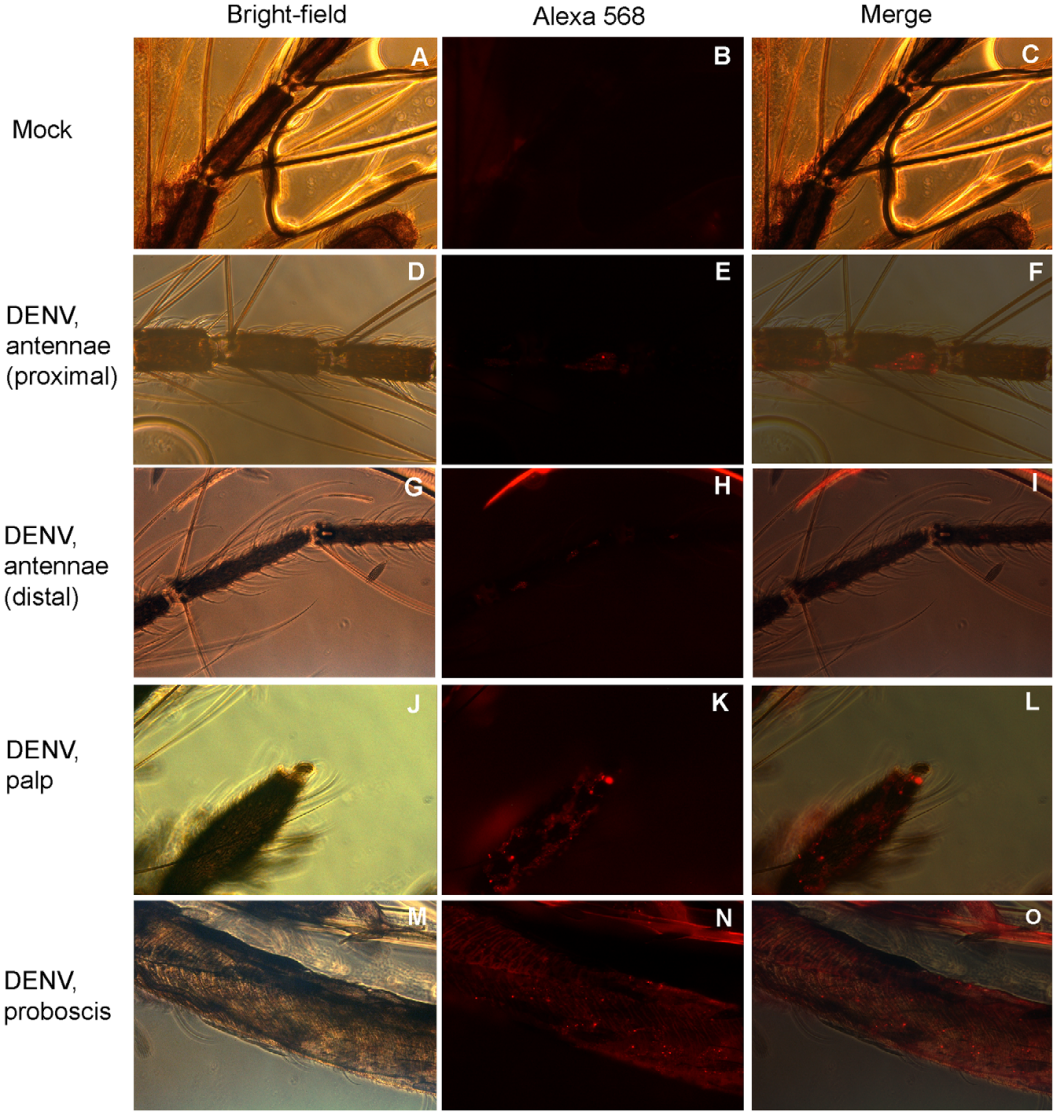
Detection of DENV infection in the chemosensory organs by immunofluorescence staining. Head squashes from mosquitoes at 14 dpbm were stained with mouse hyperimmune ascitic fluid to DENV and an AlexaFluor568-conjugated anti-mouse antibody. (**A–C**) Uninfected antennae, (**D–F**) Infected antennae at segments 2–4, (**G–I**) Infected antennae at segments 9–11, (**J–L**) Infected maxillary palp, (**M–O**) Infected proboscis. Red, DENV antigen.

To test the hypothesis that DENV infects the antennae and as such can influence OBP transcript abundance, immunofluorescent staining was first performed on head squashes of orally-infected mosquitoes at 14 dpbm. DENV-infected cells were clearly present in the antennae of infected mosquitoes but not in mock-infected controls (Figure 5). Female *A. aegypti* antennae consist of 13 flagellar segments; DENV-specific labeling was detected throughout the antennae but was stronger in the proximal segments (Figure 5D–I). DENV labeling was also detected in the maxillary palps and the proboscis (Figure 5J–O). Additionally, we also detected DENV by quantitative RT-PCR in the antennae and palps at 10 and 14 dpbm. Relative DENV loads were significantly higher at 14 dpbm compared to 10 dpbm, indicating that virus actively replicates in the chemosensory organs (Figure 4B).

We next compared OBP transcript abundance in the chemosensory organs (antennae, palps and proboscis) of DENV- and mock-infected mosquitoes. While OBP22 transcript abundance was not affected by DENV infection, OBP10 transcripts were enriched by almost 2.5-fold at 14 dpbm (Figure 4C).

Insect ORs form heteromeric complexes consisting of a conventional OR and a highly conserved universal co-receptor, termed OR co-receptor (Orco) [46]. Orco is required for trafficking of OR/Orco complexes to the sensory cilia where signal transduction occurs, and is essential for OR-mediated chemosensation *in vivo* [47]. We found that *A. aegypti* Orco (Aaeg\Orco) transcripts were enriched by approximately two-fold in the chemosensory organs of DENV-infected mosquitoes as compared to mock-infected mosquitoes at 14 dpbm (Figure 4C).

### Effect of DENV infection on mosquito blood-feeding behavior

**Figure 6:**
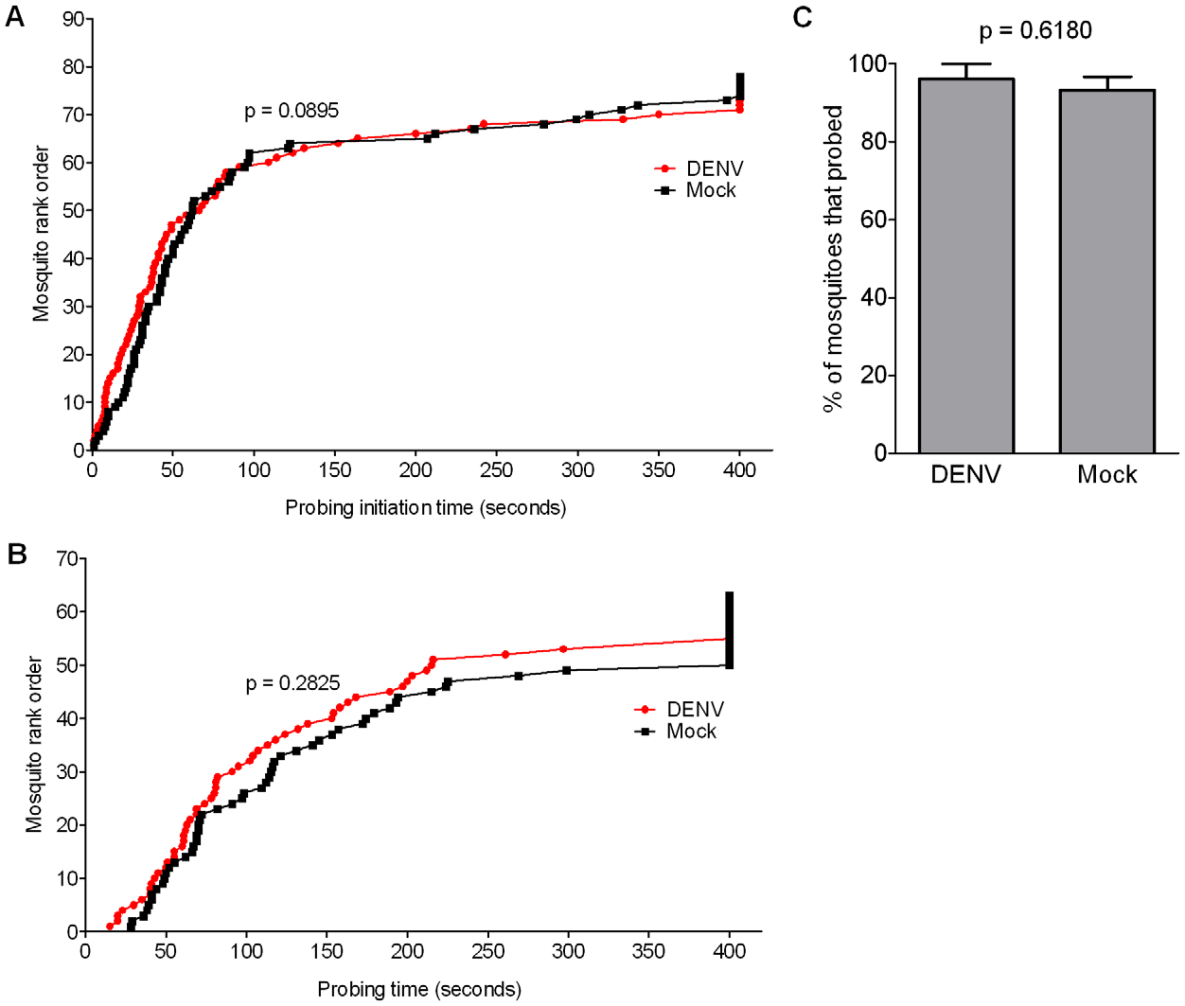
Effect of DENV infection on mosquito blood-feeding behavior. A behavioral feeding assay was performed on DENV- and mock-infected mosquitoes at 14 dpbm. Mosquitoes were offered an anesthetized mouse and observed for 400 seconds. The following parameters were measured and are represented here: (**A**) Probing initiation time; (**B**) Probing time; and (**C**) Probing propensity. The data are a pool of three biological replicates, and p values were determined with the Mann-Whitney U test.

The behavioral and gene expression data presented above suggest that DENV infection may heighten the chemosensory abilities of mosquitoes, rendering them more efficient at bloodmeal acquisition. To test this hypothesis, we compared the blood-feeding behavior of DENV- and mock-infected mosquitoes at 14 dpbm. Slightly shorter probing initiation and probing times were observed for DENV-infected mosquitoes compared to mock-infected mosquitoes, but these differences were not statistically significant (Figure 6A, B). Furthermore, we did not observe changes in probing propensity upon DENV infection (Figure 6C).

## Discussion

We have used genome-wide microarray analyses to characterize the naïve and DENV-infected *A. aegypti* salivary gland transcriptomes, and to identify candidate genes with potential roles in controlling DENV replication or mosquito feeding behavior. RNAi-mediated gene silencing in conjunction with infection assays revealed three genes that modulate DENV replication in the salivary gland, and two olfaction-related genes that modulate mosquito host-seeking and blood-feeding behavior.

DENV induced 130 and repressed 17 transcripts in the salivary gland at 14 dpbm, indicating that significant molecular and biochemical changes are induced in this organ by infection. In contrast, DENV infection appeared to have an overall negative effect on transcript abundance in the mosquito carcass, repressing more than half of the 135 differentially represented transcripts in this compartment at the same time point. The carcass response to infection at this late stage of infection was also subdued compared to what we have previously observed at 10 dpbm [14], suggesting that the transcriptional response is being negatively regulated after an initial induction phase. Furthermore, the salivary gland infection-responsive genes were largely distinct from those in the carcass at the same time point (14 dpbm), as well as from those regulated at 10 dpbm in both carcass and midgut [14], suggesting unique host-pathogen interactions. It is of course possible that some of the many tissues and cell types of the carcass may have displayed greater changes in transcript abundance that were not detected because of a dilution with the transcripts of other compartments.

As has been previously reported [6], [7], the naïve salivary gland was enriched for transcripts involved in the digestion of blood and sugar meals, and for those that play anti-hemostatic and anti-inflammatory roles during bloodmeal acquisition. In addition, the gland was also enriched for numerous transcripts with immunity-related functions, suggesting that this organ is capable of mounting an immune response against both vertebrate pathogens and microbes encountered during feeding. An unexpected finding was the large number of transcripts with putative chemosensory roles, many of which were found to be enriched in the naïve gland compared to the carcass tissue, suggesting an as yet uncharacterized function for these molecules in mosquito saliva.

Despite their high abundance in the antennae and maxillary palps, mosquito OBPs are not exclusively expressed in olfactory tissue. OBPs have previously been detected in the salivary gland, as well as in other body compartments such as the proboscis, thoracic spiracles, midgut, and even *A. aegypti* semen [6], [26], [27], [48]–[50]. Indeed, a screen of *Culex quinquefasciatus* OBPs revealed that only a minority are transcriptionally expressed solely in olfactory tissue [51]. In *A. gambiae*, a number of OBP transcripts are enriched in the bodies of males and females relative to olfactory tissue, without the coordinate expression of chemoreceptors [52]. Taken together, these studies suggest multiple ligand-binding roles for OBPs beyond olfaction. Insect OBPs and chemosensory proteins (CSPs) have been isolated in non-chemosensory organs complexed with endogenous ligands [53], [54], suggesting roles similar to those of vertebrate OBPs which deliver hydrophobic pheromones to the environment in urine or saliva.

The intimate association of the salivary gland and the proboscis raises the possibility that salivary gland-expressed OBPs and ORs may function in gustatory-related roles in the proboscis during blood- and sugar-feeding. Indeed, OBPs and ORs have been detected in the mosquito and fly proboscis [48], [55]–[57], as well as in other gustatory tissues [58], suggesting dual roles for these molecules in olfaction and taste. In addition, although the proboscis is primarily a gustatory organ, it also responds to olfactory stimuli, and OR neurons extend from the proboscis into the antennal lobes of the brain, suggesting that the proboscis may be involved in olfactory processes that are important at close proximity to the host, such as alighting, probing and blood-feeding [59]. We speculate that chemosensory molecules secreted in saliva could coat the proboscis and facilitate tasting, although there is as yet no evidence for this process.

RNAi-mediated gene silencing assays were performed to identify genes that may modulate DENV replication in the salivary gland as a mosquito defense response. The silencing of a cathepsin B gene significantly reduced salivary gland DENV titers, while silencing of a putative cystatin significantly increased DENV titers in the salivary gland. These genes could potentially play roles in apoptosis: cathepsins are lysosomal cysteine proteases that trigger apoptosis through both caspase-dependent and –independent pathways, probably by leaking or translocating from the lysosome into the cytosol [37], [60], while cystatins are cysteine protease inhibitors that may regulate the activity of pro-apoptotic caspases and cathepsins [41], [42]. While DENV-induced cytopathology has not been observed in *A. aegypti* [61], [62], West Nile virus (WNV) (also a mosquito-borne flavivirus) infections have been reported to cause cytopathology with features of apoptosis in both salivary glands and midguts of *Culex* mosquitoes [28], [29], [63], although it should be noted that WNV tends to replicate to higher titers than DENV [29], [64].

A possible hypothesis for our observations is that apoptosis of infected cells facilitates the cell-to-cell spread of DENV in the salivary gland. A separate but related possibility is that infected cells that produce dsRNA triggers of RNAi are removed from the population by apoptosis, thereby facilitating infection by the virus. Inhibition of apoptosis (by cathepsin B silencing for example) would preserve these cells, maintaining dsRNA production and impairing DENV replication through RNAi. Since silencing a cysteine protease (cathepsin B) and a cysteine protease inhibitor (the putative cystatin) resulted in opposite effects on virus titers, it is tempting to speculate that these two genes are involved in the same process, but further studies are obviously required to test this hypothesis.

Silencing of a gene encoding a hypothetical protein containing ankyrin repeats resulted in significantly elevated salivary gland DENV titers. Ankyrin repeats mediate protein-protein interactions and are present in immune-related proteins such as the IkB inhibitory domain of Rel2, the NFkB-like transcription factor of the mosquito IMD immune signaling pathway. This protein lacks a predicted signal peptide, and could act intracellularly to regulate immune signaling.

DENV infection induced OBP10 and OBP22 transcripts in the salivary gland, a finding that surprised and intrigued us. OBPs facilitate the olfactory processes of host-seeking and probing, which mosquitoes rely on for bloodmeal acquisition. Since DENV transmission also relies on these same processes, we investigated the possibility that these OBPs influence feeding behavior. Indeed, silencing of these OBP genes reduced the percentage of mosquitoes that probed on mice, and also increased probing initiation and probing times, indicating less efficient feeding behavior. To our knowledge, this is the first observation of potential arbovirus modulation of mosquito feeding behavior through chemosensory-related molecules.

We could not conclusively determine if OBP gene silencing in the salivary glands or in the chemosensory organs was responsible for this impaired feeding behavior. OBP silencing efficiency in the salivary gland was much higher and more consistent than in the antennae and maxillary palps, suggesting that silencing in the salivary glands might be responsible for at least part of the observed feeding impairment. More efficient gene silencing in *A. aegypti* antennae has been achieved through thoracic injection of dsRNA into pupae [65], as well as with a double subgenomic Sindbis virus expression system [66]; these methods may be useful for elucidating the location of action of these OBPs.

Since both probing initiation time (a rough measure of host-seeking behavior, associated with antennal function) and probing time (associated with salivary proteins that inhibit hemostasis and inflammation) were negatively affected by gene silencing, both compartments could potentially be involved. In consideration of this, we provide evidence that DENV successfully infects and replicates in the female *A. aegypti* antennae and maxillary palps, and that transcripts of one of the OBPs (OBP10) also increases in the chemosensory organs upon DENV infection. In addition, increased transcript abundance of Aaeg\Orco, the universal OR co-receptor which is essential for OR-mediated chemosensation, was also observed in the chemosensory apparatus. The significance of this is unclear, but may indicate an overall increase in OR/Orco complex abundance and chemosensory-related signal transduction during DENV infection.

Insect OBPs are quite diverse, and more than 60 members of this family have been found in the *A. aegypti* genome [24]. OBPs 10 and 22 are both “classic” OBPs containing a highly conserved pattern of six cysteine residues, and share 44% protein sequence identity but almost no nucleotide sequence similarity. Neither of these OBPs is exclusively expressed in olfactory tissue. OBP10 is male-enriched, increases with mosquito age, and (in addition to the antennae and palps) is expressed in the wings, legs, and proboscis [58]. Since gustatory sensilla are present on these tissues [67], this OBP could be involved in taste perception mediated through these organs. OBP22 has been detected in *A. aegypti* semen, and is transferred to the spermathecae of mated females [48], [49], perhaps indicating roles in pheromone binding and delivery. OBP22 is also expressed in thoracic tracheal spiracles, suggesting roles in respiration, and in the proboscis [48]. The expression patterns of these OBPs make it difficult to pinpoint their mode and location of action, but suggest that they could fulfill multiple functions.

Our data imply that viral induction of OBPs could facilitate mosquito host-seeking and/or probing behavior, and thus at least theoretically increase transmission efficiency. DENV readily infects the mosquito brain, nervous system [4], [68], and, as we show here, the chemosensory apparatus, making such behavioral modulation plausible. A number of groups have reported changes in locomotor activity and metabolism in *A. aegypti* infected with various pathogens or symbionts [69]–[71]; specifically, DENV-infected *A. aegypti* displayed an increase in locomotor activity [71], perhaps suggesting an increased ability to seek out hosts. However, we found no significant differences between the feeding behavior of DENV-infected and mock-infected mosquitoes, although a small shift towards shorter probing initiation and probing times was observed in infected insects. As behavioral experiments are sensitive to numerous environmental variables that are difficult to control in a laboratory setting, this does not rule out the hypothesis that DENV modulates mosquito feeding behavior through the regulation of chemosensory transcripts. In the field, mosquitoes must be able to locate hosts over longer distances and this feature cannot be effectively replicated in the laboratory; small differences in feeding behavior may thus have greater consequences on host-seeking under such conditions. In addition, mosquito defense mechanisms against arboviral infections can carry fitness costs [72]. The high level of DENV infection achieved under our experimental conditions alters many physiological processes other than chemosensation, such as energy metabolism, immunity, and stress responses, any of which could also affect feeding behavior, thereby counteracting the direct effect exerted on the chemosensory system.

Apart from our data, the effect of DENV infection on mosquito feeding behavior has been previously studied, with conflicting results: one study found no effect of infection status on feeding behavior [73], while another observed longer probing times in DENV-infected mosquitoes [68]. In agreement with the latter study, infection has also been found to increase intradermal probing times for several other pathogen-vector combinations [74]–[76], and it is thought that this allows more time for inoculation of the pathogen into the vertebrate host. In these scenarios, we speculate that an up-regulation of chemosensory-related transcripts may be a result of an attempt to compensate for this less efficient feeding behavior.

Our transcriptomic analysis suggests novel and uncharacterized roles for many genes in salivary gland function and response to pathogens. In addition, DENV infection in the salivary gland not only regulates genes that modulate virus replication, but also genes that potentially affect bloodmeal acquisition (and hence DENV transmission) by modifying mosquito host-seeking or probing behavior. Further characterization of these genes will yield a clearer picture of these reciprocal host-pathogen interactions in this poorly-studied organ.

## Materials and Methods

### Ethics statement

This study was carried out in strict accordance with the recommendations in the Guide for the Care and Use of Laboratory Animals of the National Institutes of Health. The protocol was approved by the Animal Care and Use Committee of the Johns Hopkins University (Permit Number: M006H300). Commercial anonymous human blood was used for dengue virus infection assays in mosquitoes, and informed consent was therefore not applicable. The Johns Hopkins School of Public Health Ethics Committee has approved this protocol.

### Mosquito rearing and cell culture

*A. aegypti* mosquitoes (Rockefeller/UGAL strain) were maintained on 10% sucrose solution at 27°C and 95% humidity with a 12 h light/dark cycle. The C6/36 *Aedes albopictus* cell line was maintained in MEM (Gibco) supplemented with 10% heat-inactivated FBS, 1% L-glutamine, 1% non-essential amino acids, and 1% penicillin-streptomycin. BHK-21 (clone 15) hamster kidney cells were maintained on DMEM (Gibco) supplemented with 10% FBS, 1% L-glutamine, 1% penicillin-streptomycin, and 5 ug/ml Plasmocin (Invivogen). C6/36 cells were incubated at 32°C and 5% CO_2_, while BHK-21 cells were incubated at 37°C and 5% CO_2_.

### DENV infections

Mosquito infections with DENV were carried out as previously described [77]. The New Guinea C (NGC) DENV-2 strain was propagated in C6/36 cells: Cells seeded to 80% confluency in 75 cm^2^ flasks were infected with virus stock at a multiplicity of infection (MOI) of 3.5, and incubated for 6 days at 32°C and 5% CO_2_. Infected cells were scraped into solution and lysed to release virus particles by repeated freezing and thawing in dry ice and a 37°C water bath. Virus suspension was mixed 1∶1 with commercial human blood and supplemented with 10% human serum. For experiments involving an uninfected control, a flask of uninfected C6/36 cells was maintained under similar conditions and used to create a naïve bloodmeal. The bloodmeal was maintained at 37°C for 30 min and then offered to mosquitoes via an artificial membrane feeding system.

### Sample collection and preparation for microarray gene expression analysis

DENV-infected and control mosquitoes were dissected at 14 days post-bloodmeal, and salivary glands and carcasses were collected and stored in Buffer RLT (Qiagen) with 1% β-mercaptoethanol. Three independent biological replicates were performed, with approximately 200 salivary glands and 20 carcasses per replicate. Total RNA was extracted from samples using the RNeasy Mini kit (Qiagen).

### Microarray gene expression analysis

The Low Input Quick Amp Labeling kit (Agilent Technologies) was used to synthesize Cy-3- or Cy-5-labeled cRNA probes from total RNA (100 ng for salivary gland samples and 200 ng for carcass samples). In addition to three biological replicates, a pseudo-replicate containing an equal amount of Cy-5-labeled probe from each experimental biological replicate was also included. Hybridizations were carried out on an Agilent-based microarray platform using custom-designed whole genome 4×44K *A. aegypti* microarrays, and arrays were scanned with an Agilent Scanner. Expression data were processed and analyzed as previously described [14], [15], [78]; in brief, background-subtracted median fluorescent values were normalized with the LOWESS normalization method, and Cy5/Cy3 ratios from replicate assays were subjected to t-tests at a significance level of p<0.05 using TIGR MIDAS and MeV software. Expression data from replicate assays were averaged with the GEPAS microarray preprocessing software (http://www.gepas.org) and logarithm (base 2)-transformed. Self-self hybridizations have been used to determine the cutoff value for the significance of gene regulation on these microarrays to 0.78 in log_2_ scale, which corresponds to 1.71-fold regulation [79]. Numeric microarray gene expression data are presented in Tables S1, S2, S3, S4.

For classification of transcripts by abundance, microarray spot hybridization fluorescence intensities were used as an indicator of transcript abundance. Spot intensity values were averaged across replicate assays for each spot, and then for each gene using the GEPAS software. Transcripts were then categorized into three categories based on mean fluorescence value (at 635 nm): high abundance (fluorescence intensity ≥5000), medium abundance (1000–5000), and low abundance (≤1000). Data are presented in Table S3.

### Gene silencing assays

RNA interference (RNAi)-mediated candidate gene silencing in mosquitoes was performed as previously described [14], [15], [80]. For gene silencing assays targeting the salivary gland, 5-day old female mosquitoes fed with DENV-supplemented blood were held until 7 dpbm, at which time they were cold-anesthetized and injected with 2 ug of dsRNA per mosquito [19]. Mosquitoes injected with dsRNA to GFP were used as controls. Salivary glands were dissected at 14 dpbm and individually stored in DMEM at −80°C until they were titrated by plaque assay. Data presented are a pool of three independent biological replicates, and p-values were determined with the Mann-Whitney U test. dsRNA was synthesized using the HiScribe T7 *in vitro* transcription kit (New England Biolabs). The primer sequences used for dsRNA synthesis are presented in Table S5, and primer sequences used to confirm gene silencing by real-time PCR are presented in Table S6.

### DENV titration by plaque assay

DENV titers in midguts and salivary glands were determined by plaque assay on BHK-21 (clone 15) cells. Individual midguts and salivary glands were homogenized in DMEM with a Bullet Blender (NextAdvance), serially diluted, and then inoculated onto cells seeded to 80% confluency in 24-well plates (100 ul per well). Plates were rocked for 15 min at room temperature, and then incubated for 45 min at 37°C and 5% CO_2_. Subsequently, 1 ml of DMEM containing 2% FBS and 0.8% methylcellulose was added to each well, and plates were incubated for 5 days at 37°C and 5% CO_2_. Plates were fixed with a methanol/acetone mixture (1∶1 volume) for >1 h at 4°C, and plaque-forming units were visualized by staining with 1% crystal violet solution for 10 min at room temperature.

### Host-seeking and probing assays

Candidate genes were silenced in 4-day old female mosquitoes by the injection of 2 ug of dsRNA per mosquito (dsRNA to GFP was used as a control), and behavioral assays were carried out at 4 days post-injection. Mosquitoes were deprived of sucrose solution overnight prior to the assay. Mosquitoes were transferred in pairs to a small cage and allowed to rest for at least 10 min before being offered an anesthetized Swiss Webster mouse. The mosquitoes were observed for a maximum of 400 seconds, and the following parameters were measured for each mosquito: a) Probing propensity (percentage of mosquitoes that probed within the fixed time period of 400 seconds); b) Probing initiation time (time from the introduction of the mouse to the time at which the mosquito begins to probe); and c) Probing time (time from the initial insertion of the mouthparts in the skin to the initial engorgement of blood; if the mosquito makes multiple probing attempts, the subsequent probing times are added to the first until blood is ingested, and the interprobing times are not included [6], [10]). Data presented are a pool of six independent biological replicates, and p-values were determined with the Mann-Whitney U test.

Behavioral assays involving DENV-infected mosquitoes were carried out at 14 dpbm. A maximum of seven mosquitoes were placed in a single cage and deprived of sucrose solution overnight. A Swiss-Webster mouse was euthanized via the administration of an overdose of ketamine, and offered to the mosquitoes. To counteract the drop in body temperature, a heat pack was placed over the mouse for the duration of the assay. Because of the larger number of mosquitoes per cage, video recordings were made during the assay to allow the experimenter to keep track of individual mosquitoes. As above, probing propensity, probing initiation time and probing time were measured.

### Real-time PCR quantification of odorant-binding protein gene expression and detection of DENV in the antennae

Quantitative RT-PCR was used to measure relative transcript abundance of odorant-binding proteins in the mosquito chemosensory apparatus following DENV infection, as well as to measure relative DENV loads in these organs. Antennae and maxillary palps were dissected from DENV- and mock-infected mosquitoes at 10 and 14 days post-bloodmeal. Total RNA was extracted from samples using the RNeasy Mini Kit (Qiagen), treated with Turbo DNase (Ambion) and reverse-transcribed with M-MLV Reverse Transcriptase (Promega) and oligo-dT20. For detection and relative quantification of DENV transcripts, total RNA was reverse-transcribed with a DENV-specific reverse primer. Real-time quantification was performed using SYBR Green PCR Master Mix and the StepOne Plus Real-Time PCR system (Applied Biosystems). Three independent biological replicates were analyzed, and technical duplicates were run for each sample. Expression values were normalized against the ribosomal gene S7. p-values were determined with the student's t test. Primers used in this assay are listed in Table S6.

### Indirect immunofluorescence assay for detection of DENV antigen

Heads were dissected from DENV- and mock-infected mosquitoes at 14 days post-bloodmeal, squashed on 3-aminopropyltriethoxysilane (APES)-treated glass slides, and fixed in acetone at 4°C overnight. Slides were incubated with mouse hyper-immune ascitic fluid specific for DENV2 (diluted 1∶1000 in PBS with 0.1% Triton X-100 and 0.2% BSA) at 4°C overnight, and washed three times in PBS. Slides were then incubated with AlexaFluor 568-conjugated goat anti-mouse IgG (Molecular Probes) for 1 hour at room temperature, and washed three times in PBS. Samples were covered with ProLong Gold Antifade with DAPI (Invitrogen), and sealed with a cover-slip and nail varnish. Slides were visualized under a Leica fluorescence microscope.

## Supporting Information

Figure S1 **Salivary gland silencing efficiencies of candidate genes in dsRNA-injected mosquitoes.** Mosquitoes were each injected with 2 ug of dsRNA to the candidate gene or to dsGFP as a control. Salivary glands were collected at 4 days post-injection. Silencing efficiencies were determined by quantitative RT-PCR, and expression values were normalized against the ribosomal gene S7. Gene name abbreviations: AnkP, ankyrin repeat-containing protein; Cyst, cystatin; SSP, salivary secreted peptide; CatB, cathepsin B.
(TIF)

Figure S2 **DENV titers in the carcasses of mosquitoes injected with dsRNA.** Effect of candidate gene knockdown on carcass DENV titers. Candidate genes were silenced in DENV-infected mosquitoes, and carcass (with salivary glands and heads removed) titers at 14 dpbm were determined by plaque assay. A pool of three biological replicates is shown. In the Mann-Whitney U test, DENV titers in candidate gene silenced-mosquitoes were not significantly different from titers in dsGFP-treated mosquitoes. Gene name abbreviations: AnkP, ankyrin repeat-containing protein; Cyst, cystatin; CatB, cathepsin B.
(TIF)

Table S1 **Log_2_-fold values and functional groups of transcripts that were significantly enriched in the naïve salivary gland relative to the carcass.** Functional group abbreviations: CS, cytoskeletal and structural; CSR, chemosensory reception; DIV, diverse functions; DIG, blood and sugar food digestive; IMM, immunity; MET, metabolism; PROT, proteolysis; RSM, redox, stress and mitochondrion; RTT, replication, transcription, and translation; TRP, transport; UKN, unknown functions.
(XLSX)

Table S2 **Spot intensities, log_2_-fold values, and functional groups of transcripts expressed in the naïve salivary gland.** Functional group abbreviations: CS, cytoskeletal and structural; CSR, chemosensory reception; DIV, diverse functions; DIG, blood and sugar food digestive; IMM, immunity; MET, metabolism; PROT, proteolysis; RSM, redox, stress and mitochondrion; RTT, replication, transcription, and translation; TRP, transport; UKN, unknown functions.
(XLSX)

Table S3 **Log_2_-fold values and functional groups of transcripts that were significantly regulated by DENV infection in the salivary gland at 14 days post-infection.** Functional group abbreviations: CS, cytoskeletal and structural; CSR, chemosensory reception; DIV, diverse functions; DIG, blood and sugar food digestive; IMM, immunity; MET, metabolism; PROT, proteolysis; RSM, redox, stress and mitochondrion; RTT, replication, transcription, and translation; TRP, transport; UKN, unknown functions.
(XLSX)

Table S4 **Log_2_-fold values and functional groups of transcripts that were significantly regulated by DENV infection in the carcass at 14 days post-infection.** Functional group abbreviations: CS, cytoskeletal and structural; CSR, chemosensory reception; DIV, diverse functions; DIG, blood and sugar food digestive; IMM, immunity; MET, metabolism; PROT, proteolysis; RSM, redox, stress and mitochondrion; RTT, replication, transcription, and translation; TRP, transport; UKN, unknown functions.
(XLSX)

Table S5 **Sequences of primers used for synthesis of double-stranded RNA.**
(DOCX)

Table S6 **Sequences of primers used for real-time PCR quantification.**
(DOCX)
