# Supplementary figures and images for: Dengue Virus Infection of the Aedes aegypti Salivary Gland and Chemosensory Apparatus Induces Genes that Modulate Infection and Blood-Feeding Behavior
Source: PLoS Pathog. 2012 Mar 29;8(3):e1002631. doi: 10.1371/journal.ppat.1002631 (PMC3315490; doi:10.1371/journal.ppat.1002631)

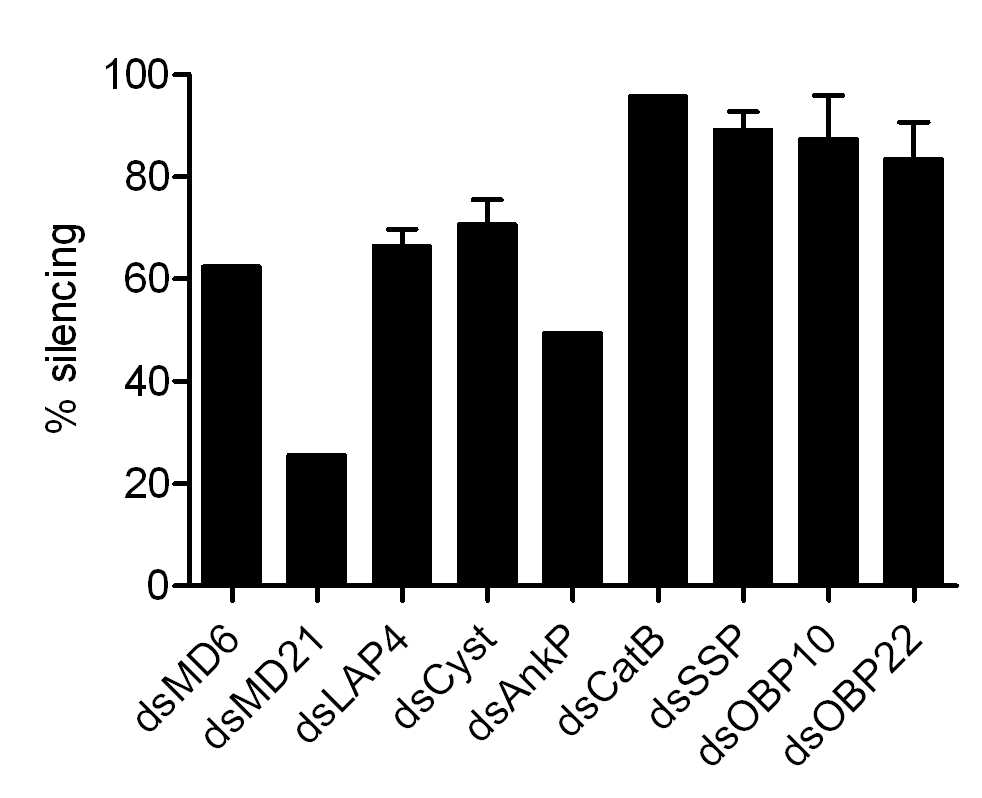

Supplement: Figure S1 — Salivary gland silencing efficiencies of candidate genes in dsRNA-injected mosquitoes. Mosquitoes were each injected with 2 ug of dsRNA to the candidate gene or to dsGFP as a control. Salivary glands were collected at 4 days post-injection. Silencing efficiencies were determined by quantitative RT-PCR, and expression values were normalized against the ribosomal gene S7. Gene name abbreviations: AnkP, ankyrin repeat-containing protein; Cyst, cystatin; SSP, salivary secreted peptide; CatB, cathepsin B. (TIF) [file ppat.1002631.s001.tif]

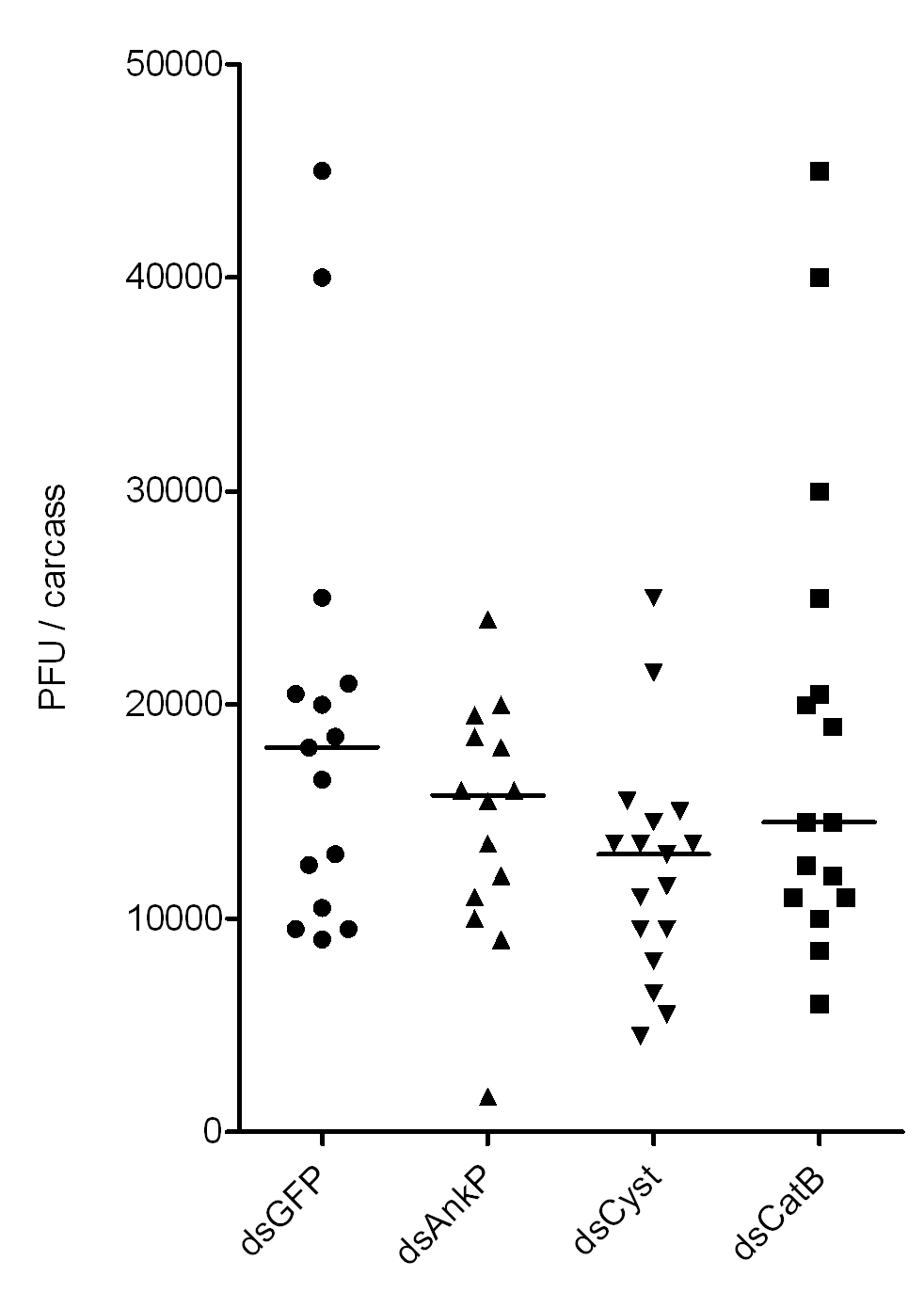

Supplement: Figure S2 — DENV titers in the carcasses of mosquitoes injected with dsRNA. Effect of candidate gene knockdown on carcass DENV titers. Candidate genes were silenced in DENV-infected mosquitoes, and carcass (with salivary glands and heads removed) titers at 14 dpbm were determined by plaque assay. A pool of three biological replicates is shown. In the Mann-Whitney U test, DENV titers in candidate gene silenced-mosquitoes were not significantly different from titers in dsGFP-treated mosquitoes. Gene name abbreviations: AnkP, ankyrin repeat-containing protein; Cyst, cystatin; CatB, cathepsin B. (TIF) [file ppat.1002631.s002.tif]
