# Supplementary material for: Dengue Virus Infection of the Aedes aegypti Salivary Gland and Chemosensory Apparatus Induces Genes that Modulate Infection and Blood-Feeding Behavior
Source: PLoS Pathog. 2012 Mar 29;8(3):e1002631. doi: 10.1371/journal.ppat.1002631 (PMC3315490; doi:10.1371/journal.ppat.1002631)
Supplement: Table S5 — Sequences of primers used for synthesis of double-stranded RNA. (DOCX) [file ppat.1002631.s007.docx]

**Table S5.** Sequences of primers used for synthesis of double-stranded RNA.

| **Gene ID** | **Name** | **Sequence** |
| --- | --- | --- |
| AAEL003728 | dsAnkprotein F | TAATACGACTCACTATAGGGCCGCAAGGACGGTCCCTCGT |
| AAEL003728 | dsAnkprotein R | TAATACGACTCACTATAGGGTGGTCCCACGCAAGTGGCAG |
| AAEL005772 | dsOBP22 F | TAATACGACTCACTATAGGGTGGCTGCCGCTGAGTTCACC |
| AAEL005772 | dsOBP22 R | TAATACGACTCACTATAGGGGAAGGCCCAGTGGCAAGCGT |
| AAEL007585 | dsCathpB F | TAATACGACTCACTATAGGGGTACTGGCCTGCTGCCAGGC |
| AAEL007585 | dsCathpB R | TAATACGACTCACTATAGGGTGGCCGAGGCAGCTGAAACG |
| AAEL007603 | dsOBP10 F | TAATACGACTCACTATAGGGACGAGCTGCACTGCATCGAAGC |
| AAEL007603 | dsOBP10 R | TAATACGACTCACTATAGGGAGCCTTGTTCTGGCGGTAGCAA |
| AAEL009760 | dsMDL21 F | TAATACGACTCACTATAGGGGTCGGACAGCTGGAGGTGCA |
| AAEL009760 | dsMDL21 R | TAATACGACTCACTATAGGGTGGCGATTGGCGACTCGACC |
| AAEL013287 | dsCystatin F | TAATACGACTCACTATAGGGTGAACGCCGAGCACCACGAC |
| AAEL013287 | dsCystatin R | TAATACGACTCACTATAGGGCTCGCCCTTGTCCTGCAGCC |
| AAEL014906 | dsLAP4 F | TAATACGACTCACTATAGGGAGCGACCTTGATCAACCAGGC |
| AAEL014906 | dsLAP4 R | TAATACGACTCACTATAGGGTGCTCGGCAAGATCCGCTCG |
| AAEL015136 | dsMDL6 F | TAATACGACTCACTATAGGGAGCTGAAACGCGGCGAGGAC |
| AAEL015136 | dsMDL6 R | TAATACGACTCACTATAGGGGCTCTGCTCCACCGGCAACC |
| AAEL017380 | dsSSP F | TAATACGACTCACTATAGGGGGCCGTAAGCTGTGCCCCAC |
| AAEL017380 | dsSSP R | TAATACGACTCACTATAGGGCCACCTCCCATGCCGGGGAA |
| NA | dsGFP F | TAATACGACTCACTATAGGATGGTGAGCAAGGGCGAGGAGCTGT |
| NA | dsGFP R | TAATACGACTCACTATAGGTTACTTGTACAGCTCGTCCATGCCG |
