# Supplementary material for: Dengue Virus Infection of the Aedes aegypti Salivary Gland and Chemosensory Apparatus Induces Genes that Modulate Infection and Blood-Feeding Behavior
Source: PLoS Pathog. 2012 Mar 29;8(3):e1002631. doi: 10.1371/journal.ppat.1002631 (PMC3315490; doi:10.1371/journal.ppat.1002631)
Supplement: Table S6 — Sequences of primers used for real-time PCR quantification. (DOCX) [file ppat.1002631.s008.docx]

**Table S6.** Sequences of primers used for real-time PCR quantification.

| **Gene ID** | **Name** | **Sequence** |
| --- | --- | --- |
| AAEL003728 | RT AnkProtein F | ATTGCTGCCACTTGCGTGGG |
| AAEL003728 | RT AnkProtein R | GTGAGGCCTCTTCGGGCGAA |
| AAEL005772 | RT OBP22 F | ACGCCGAGGAAGTTCGCACC |
| AAEL005772 | RT OBP22 R | TGCTAGCCTTGATCAGCGACAGG |
| AAEL007585 | RT CathpB F | GTGGCCAACTCGTGGGGTCG |
| AAEL007585 | RT CathpB R | AAGTTGGGCAGACCCGCGTG |
| AAEL007603 | RT OBP10 F | TATGAGAGGGGTCCATTCCGCGG |
| AAEL007603 | RT OBP10 R | ATCGACTGATCGGGGCTGGCTA |
| AAEL009496 | RT S7 F | GCAGACCACCATTGAACACA |
| AAEL009496 | RT S7 R | CACGTCCGGTCAGCTTCTTG |
| AAEL009760 | RT MDL21 F | AGCGAAATTGACGTTGCTGATCGT |
| AAEL009760 | RT MDL21 R | CGGGCAATCACCTTGGCGGT |
| AAEL013287 | RT Cystatin F | CGGAGGAGTGCATCCGACGG |
| AAEL013287 | RT Cystatin R | CCTGCTGGGTGGACCGCAAA |
| AAEL014906 | RT LAP4 F | GCTGCCCTGCGGAAAGGAGAC |
| AAEL014906 | RT LAP4 R | TGTCGGCCACATTCAACGTCTTCA |
| AAEL015136 | RT MDL6 F | CCGCTAGTGTTCGCCGACGT |
| AAEL015136 | RT MDL6 R | GTCCTCGCCGCGTTTCAGCT |
| AAEL017380 | RT SSP F | GGCATGGGAGCTTCGTCTGC |
| AAEL017380 | RT SSP R | TGTGCTCCAGCGTTTGCGTTCG |
| NA | DENV F | TCAATATGCTGAAACGCGAGAGAAACCG |
| NA | DENV R | CGCCACAAGGGCCATGAACAGTTT |
